# Effect of Constant Inflammation on *In Vitro* Expanded Adipose-derived Mesenchymal Stromal Cells

**DOI:** 10.1007/s12015-025-10906-8

**Published:** 2025-06-05

**Authors:** Marina Ramírez Galera, Tu Hu, Lisa Harth, Mariana Bronze, Lea Munthe-Fog, Jesper Svalgaard, Anders Woetmann

**Affiliations:** 1 The LEO Foundation Skin Immunology Research Center, Department of Immunology and Microbiology, Faculty of Health and Medical Sciences, University of Copenhagen, Copenhagen, Denmark; 2 Center for Vaccine Research, Department of Infectious Disease Immunology, Statens Serum Institut, Copenhagen, Denmark; 3 Celcore ApS, Soeborg, Denmark

**Keywords:** IFNγ, Constant inflammation, Mesenchymal stem cells, Adipose, Bulk-RNA sequencing, Immunosuppression

## Abstract

Human adipose-derived mesenchymal stromal cells (AD-MSCs) are known for their immunomodulatory responses to inflammation, a key trait for their therapeutic use in tissue injury and chronic inflammatory diseases. However, the effects of constant inflammation on their transcriptomic profile, proliferative capacity, and immunosuppressive potential remain largely unexplored. To investigate this, we evaluated the in vitro response of human AD-MSCs from three donors expanded across seven passages under, non-inflamed (DMEM), acute (IFNγ-24 h) and constant (IFNγ-C) inflammatory conditions. Bulk RNA-sequencing results demonstrated that AD-MSCs respond significantly to inflammatory stimuli. Both acute and constant inflammation induced extensive transcriptomic alterations, with differentially expressed genes related to cell cycle regulation, DNA replication, metabolism, and immunomodulation. Notably, constant inflammation was associated with a transcriptomic shift toward oxidative phosphorylation (OXPHOS). Moreover, microscopy analysis revealed a significant reduction in AD-MSCs proliferative capacity under constant inflammation, as evidenced by lower cumulative population doubling and the appearance of senescence‐like morphological changes in early passages when compared to non-inflamed (DMEM) and acute inflammatory (IFNγ‐24 h) conditions. Despite proliferation being impaired, co‐culture assays with PBMCs demonstrated that under constant inflammation AD-MSCs immunosuppressive potential was maintained in early and late passages. Therefore, our results provide novel insights into the dual effects of constant inflammation on AD-MSCs, impairing cell proliferation while maintaining their immunosuppressive capacity. Understanding AD-MSCs’ behaviour in chronically inflamed microenvironments is essential for optimizing AD-MSCs' clinical applications.

**Graphical Abstract:**

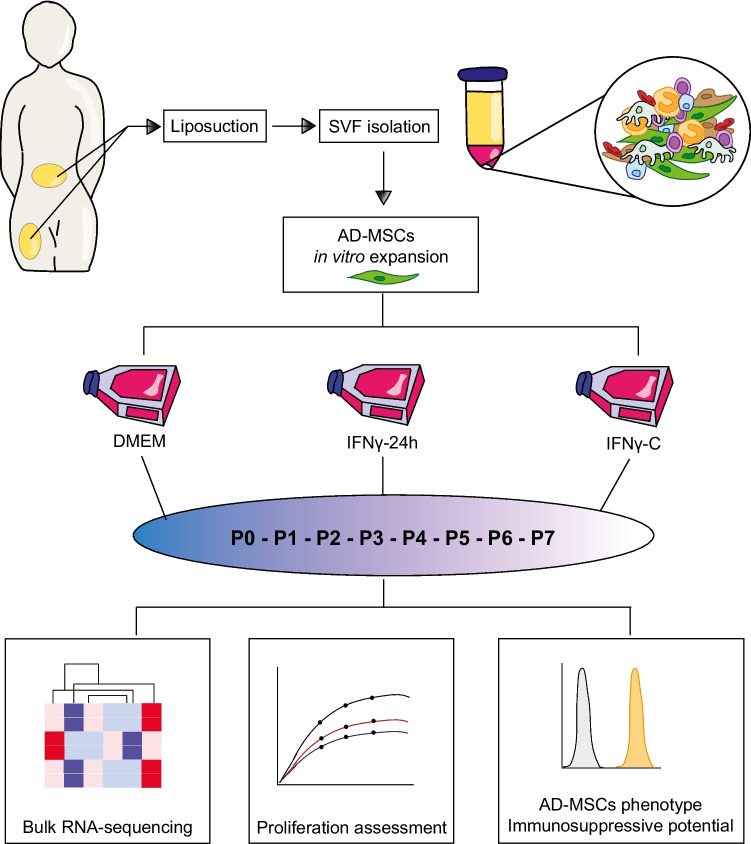

**Supplementary Information:**

The online version contains supplementary material available at 10.1007/s12015-025-10906-8.

## Introduction

Adipose-derived mesenchymal stromal cells (AD-MSCs) are being used in many clinical trials due to their regenerative and immunomodulatory properties, particularly in the context of chronic inflammatory diseases [1, 2]. According to the International society for Cell Therapy (ISCT), mesenchymal stromal cells (MSCs) are defined as non-hematopoietic stem cells that can derive from bone marrow, adipose tissue, umbilical cord and other sources from autologous or allogeneic donors [3]. They are characterised phenotypically by extracellular marker expression (positive for CD90, CD73 and CD105 while negative for hematopoietic and endothelial markers like CD45 and CD34), plastic adherence during in vitro culture and the ability to differentiate into osteogenic, chondrogenic and adipocyte cell lineages [3, 4].

Many studies have shown the potential use of MSCs in treating chronic inflammatory conditions, emphasizing their ability to modulate immune responses and promote tissue regeneration [1, 5]. Importantly, cell therapies using MSCs require the administration of high numbers of cells per patient, which requires in vitro expansion [6]. During the expansion, MSCs undergo multiple cell divisions and passages and as a result, the risk of replicative senescence increases [7]. Extensive research has characterized the impact of in vitro passaging on MSCs, highlighting alterations in their proliferation, differentiation potential, and secretory profiles [8, 9]. Similarly, inflammatory cues have been shown to modulate MSCs function, enhancing their therapeutic efficacy and immunosuppressive capabilities [10–15]. Despite these promising findings, optimising MSC-based therapies requires a deeper understanding of how chronic inflammation influences AD-MSCs at the molecular and cellular level. In chronic inflammatory diseases, administered AD-MSCs are exposed to a tissue microenvironment characterised by constant exposure to pro-inflammatory cytokines, sustained immune cell activation, and oxidative stress. Currently, the effects of constant inflammation on AD-MSCs remain largely unexplored and addressing this gap is crucial for improving treatment outcomes and refining MSC-based therapeutic strategies. In this study, we aimed to understand how constant inflammation affects AD-MSCs. Particularly, understanding how their transcriptional landscape is shaped and whether AD-MSCs are able to replicate, differentiate and maintain their immunosuppressive potential under such condition. Our findings provide essential insights into the long-term behaviour of AD-MSCs under constant inflammatory stress and contribute to the broader knowledge of their role in chronic disease environments.

## Materials and Methods

### Cell Isolation and Culture

Adipose tissue was collected from otherwise discarded material from elective cosmetic procedures and processed to isolate the stromal vascular fraction (SVF). Lipoaspirates were washed with sterile phosphate-buffered saline (PBS) to remove red blood cells and debris. Washed adipose tissue was enzymatically digested using 1 mg/mL collagenase type I (Invitrogen), 10 mg/mL bovine serum albumin (BSA) (Sigma-Aldrich), and 2 mM CaCl₂ in a ratio of 1 mL PBS per 3 mL lipoaspirate. Digestion was performed at 37 °C for 45 min with gentle agitation. The digested suspension was centrifuged at 600 g for 10 min at room temperature, and the supernatant was discarded. The resulting cell pellet, containing the SVF, was resuspended in a growth medium consisting of Dulbecco's Modified Eagle’s Medium (DMEM) low glucose (Thermo Fisher Scientific) supplemented with 10% pooled human platelet lysate (pHPL), 2 IU/mL heparin (preservative-free) (StemCell Technologies), and 100 μg/mL streptomycin and 100 U/mL penicillin (Invitrogen, Thermo Fisher Scientific).

SVF cells were seeded at a density of 10,000 cells/cm^2^ in T-175 culture flasks (NUNC, Thermo Fisher Scientific) and maintained at 37 °C and 5% CO_2_ in a humidified incubator. The initially seeded SVF fraction was designated as P0 (passage 0); subsequent passages were designated as P1-P7. Between passages, media was changed every 3 days. The cells were harvested after approximately 7 days in culture (37ºC, 5% CO_2_ incubator), when reaching approximately 80% confluency. Briefly, cells were harvested by washing with PBS, followed by detachment using trypsinization TrypLE Express (Gibco), and subsequent neutralization by adding culture media. The harvested cells were used for cell counting, RNA isolation, and cryopreservation for downstream applications, including flow cytometry, co-culture, and differentiation assays. For morphology assessment, cells were seeded at 2,000 cells/cm^2^ in flat-bottom 96-well plates. For IFNγ treatments, cells were exposed to 25 ng/mL IFNγ (R&D Systems) either continuously (IFNγ-C) or for 24 h prior to harvesting (IFNγ−24 h).

AD-MSCs were cryopreserved by using 1:1 of the pre-cooled cryomedia ((DMEM 10% DMSO, 5% Human Albumin and 1% Heparin). The cryotubes were frozen at −80ºC (Mr Frosty, CorningR CoolCellTM) and transferred to liquid nitrogen 24 h later. When needed, cryopreserved cells were thawed at 37 °C and transferred into culture media.

### Cumulative Population Doubling

Cells harvested from the flasks were counted using a TC20™ Automated Cell Counter (Bio-Rad). Live cell count was used to calculate population doubling (PD) and cumulative population doubling (cumPD). PD for each passage was determined using the formula PD = (log(N_f)—log(N_i))/log(2) where Nf = final viable cell count at passage and Ni = initial seeded cell number. Cumulative population doubling cumPD = Σ (PD_i) for i = 1 to n where PD_i = population doubling at each passage n = total number of passages. The total time in culture for each passage was recorded, and cumulative PD values were plotted over time (days in culture) to compare proliferation trends among conditions. For statistical analysis, a two-way ANOVA was performed, followed by Tukey’s multiple comparison test (ordinary) to assess differences between conditions. The significance threshold was set at α = 0.05, and data are reported as mean ± standard deviation (SD) from at least three independent experiments.

### Flow Cytometry: AD-MSCs Phenotype

Thawed cells were centrifuged for 5 min at 300 × g. First, the cell pellet was resuspended in FACS buffer (PBS and 2 mM EDTA (Invitrogen, Termo Fisher Scientific)). Then Fc-block antibody (Miltenyi Biotec) was added and incubated for 10 min on ice. For membrane bound extracellular markers the antibody mix contained the following antibodies all from BD Biosciences except for Fixable viability dye (FVD) that was purchased from Invitrogen™: CD73 (BUV395; #742636), CD105 (AF647; #568987), CD45 (BUV737; #748719), CD34 (BV605; #745105), CD31 (PE-Cy7; #563651), CD90 (BV480; #746286), FVD (efluor780; # 65–0865-14), CD274 (BV789; #563739) and HLADR (BV711; # 740784).

Following 30 min incubation at 4ºC, the cells were washed with FACS buffer.

and were resuspended in BD Fixation/Permeabilization™ solution and kept at 4ºC for 20 min. Cells were then washed twice with BD PermWash™ buffer, resuspended in BD PermWash™ buffer, added Fc-block antibody and incubated for 5 min at 4ºC. IDO1-BV421 (BD Biosciences #567863) was added to the cells and kept for 30 min on ice in the dark. The cells were washed twice with BD PermWash™ buffer and resuspended in FACS buffer and analysed on a LSRFortessa™ X-20 flow cytometer (BD Biosciences) and accompanying software (FACS Diva, BD Biosciences). Further analysis was performed using FlowJo software version 10.10.0.

### PBMCs Isolation

Human PBMCs were isolated from 3 buffy coats obtained from Department of Clinical Immunology, Rigshospitalet, Denmark and donated by consenting, anonymized healthy individuals in accordance with Danish Transfusion Medical Standards. To isolate PBMCs, the buffy coats were diluted with phosphate-buffered saline (PBS) containing 2% FBS (Biological Industries) and 1 mM EDTA and carefully layered onto LymphoPrep™ density gradient medium (ProteoGenix) in SepMate™ tubes (StemCell Technologies, 50 ml) with an inert porous membrane. The samples were centrifuged at 1200 g for 20 min at room temperature. Following this, the PBMC layer was collected, and two wash steps in PBS with 2%FBS were performed with centrifugation at 300 g for 8 min and 5 min at room temperature. Before the final centrifugation, the PBMCs were filtered through a 70 µm cell strainer. Filtered cells were cryopreserved in freezing media 1:1 (RPMI 20% FBS 10% DMSO (Sigma-Aldrich)).

### Co-culture and Cell Trace CFSE Staining

Three different donors of AD-MSCs form 3 different treatments (DMEM, IFNγ−24 h and IFNγ-C) were thawed and diluted 1:10 in pre-warmed DMEM supplemented with 10% HPL and 1 × penicillin–streptomycin. The cells were centrifuged at 200 g for 10 min at room temperature and resuspended in DMEM. After counting, 20,000 cells/well were seeded in 96-well round bottom plate (Corning). AD-MSCs were incubated at 37 °C with 5% CO_2_ overnight to allow cell adherence to the well.

The next day, PBMCs were thawed and diluted 1:10 in pre-warmed RPMI-1640 supplemented with 10% FBS and 1 × penicillin–streptomycin. The cells were incubated at 37 °C with 5% CO_2_ for 1 h for recovery. After recovery, cells were centrifuged at 300xg for 5 min at room temperature, and Cell Trace CFSE (Thermo Fisher Scientific) staining was performed by resuspending 20 million cells in 2 ml PBS with 2.5% FBS to a final concentration of 5 µM CFSE. The cells were vortexed briefly and incubated at 37 °C for 10 min. Following incubation, the cells were washed three times with 5% FBS in PBS, resuspended in RPMI 10% FBS and 100 μg/mL streptomycin and 100 U/mL penicillin, and counted 1 × 10^5^ CFSE-labelled cells to seed in wells with AD-MSCs (co-culture) and without AD-MSCs (mono-culture) to track cell proliferation in 96-well round bottom plate. 1 × 10^5^ unstained PBMCs were also counted and seeded in different wells containing AD-MSCs and without for unstained control assessment. Prior to seeding the PBMCs, DMEM was removed from the wells containing AD-MSCs and washed with PBS. PBMCs were added in a final volume of 200 µl, with or without 5 µg/ml PHA-L (Sigma-Aldrich). Cells were incubated for 5 days, with media changes on day 3. At day 5 cells were harvested for flow cytometry. The plate was centrifuged at 300xg for 5 min, and the supernatant was discarded. A volume of 200 µL of FACS buffer (10% FBS, 1 mM EDTA, 0.5% sodium azide (Fagron Nordic A/S) in PBS) was added to each well, and the plates were refrigerated at 4 °C for 10 min. The cells were homogenized and centrifuged again at 300 g for 5 min. The pellet was resuspended, and replicates were pooled together. The plate was centrifuged again at 300 g for 5 min. The cells were resuspended in 50 µL per well containing an Fc blocker and incubated for 10 min at 4 °C. Next, 50 µL of antibody cocktail, including markers for CD3 (PerCpCy5.5; BD # 560,835), CD4 (BUV395; BD # 563,550), CD8 (PE; BD # 555,635), FVD (efluor780; # Invitrogen™ #65–0865-14), CD25 (BV421; Biolegend # 356,114), and CD69 (BUV496; BD #750,214), was added to each well and incubated for 30 min at 4 °C. After incubation, 100 µL of FACS buffer was added to each well, and the plate was centrifuged for 5 min at 300 g. The cells were resuspended in 200 µL of FACS buffer and ran on a LSRFortessa™ X-20 flow cytometer with FACS Diva software. Downstream analysis was performed with FlowJo software.

### Bulk-RNAseq and Bioinformatics Analysis

RNA was purified using Qiagen RNeasy Mini Kit (Qiagen), according to manufacturer’s protocol. RNA purity was validated using a NanoDrop Spectrophotometer with A260/A280 and A260/A230 minimum criteria of 2–2.2. Samples were frozen at −80 °C until sent for sequencing to BGI Europe Genome Center (COBIS, Copenhagen) for 100-bp paired-end sequencing. Raw reads were processed using the nf-core/rnaseq pipeline (v3.11.1) (https://doi.org/10.5281/zenodo.1400710) implemented in Nextflow (10.1038/nbt.3820). Briefly, reads were assessed for quality with FastQC (http://www.bioinformatics.babraham.ac.uk/projects/fastqc/), trimmed using Fastp (10.1093/bioinformatics/bty560.) and pseudoaligned to the GRCh38 reference genome (Ensembl v109) with Salmon (10.1038/nmeth.4197). The resulting data were imported into R (v4.4.0) as a SummarizedExperiment object for downstream analyses. Non-coding genes were excluded based on gene annotations queried from BioMart (10.1038/nprot.2009.97). Count normalization and variance correction were performed using functions from the tidybulk (10.1186/s13059-020-02233-7) and tidySummarizedExperiment frameworks (tidybulk:scale_abundance) (https://github.com/stemangiola/tidySummarizedExperiment).

Exploratory data analysis and visualization. Principal component analysis (PCA) was conducted on the top 500 most variable genes to visualize overall transcriptional variation. The ComplexHeatmap (https://doi.org/10.1093/bioinformatics/btw313) R package was used to generate heat maps of z-scaled expression values for the top 75 most variable genes, and highly variable genes were identified using tidybulk::keep_variable.

Differential gene expression analysis. Differentially expressed genes were identified using DESeq2 (10.1186/s13059-014-0550-8). Genes with an absolute log_2 fold change greater than 1 and an adjusted P value below 0.05 were defined as significantly differentially expressed.

Variance partition analysis. To evaluate the contribution of donor, treatment and passage to gene expression variability, the variancePartition package (10.1101/2023.03.17.533005) was used. The function fitExtractVarPartModel was applied to log_2-transformed counts, modeling each factor as a random effect.

Pathway analysis. Gene set variation analysis (GSVA) was performed on the normalized expression data using the GSVA package (10.1186/1471-2105-14-7).

Detailed parameters and the complete reproducible workflow are available on GitHub (https://github.com/tuhulab/stromal-cell-in-vitro-culture).

### Differentiation Assays

ASCs were differentiated into adipocytes and osteocytes in separate 96 well plates, according to manufacturer’s instructions (StemPro Differentiation kit, Gibco, Thermo Fisher Scientific). A density of 1 × 10^4^ viable cells/cm^2^ were seeded and pre-warmed differentiation media was refed every three days for 14 days (adipogenesis), and 21 days (osteogenesis). On the last day of cell culture, the cells were fixed with 4% formaldehyde (ChemCruz, Santa Cruz Biotechnologies) solution for 10 min and specific colorimetric stainings were performed.

In the adipogenesis plate, 60% isopropanol (Sigma-Aldrich) was added and removed after 5 min. Then, Oil Red working solution (1:3 dilution of Oil Red (Sigma-Aldrich) (stock solution (0,5% Oil Red solution diluted to 0,3% in 99% isopropanol)) and distilled water) was added and incubated for 5 more minutes, being removed with rinsing water. In the osteogenesis plate, filtered Alizarin Red solution (Sigma-Aldrich) (2 g Alizarin Red in 90 mL distilled water with final pH of 4,1–4,3) was added to the wells, incubated for 15 min and washed 4 times with distilled water.

### Statistical Analysis

Statistical analyses for cumPD and flow cytometry analysis were conducted using GraphPad Prism version 10. Data were obtained from experiments performed using three independent AD-MSCs donors as well as three independent PBMCs donors. Two-way analysis of variance (two-way ANOVA) was applied to evaluate the effects of the treatment condition and passage. Error bars in all graphs represent the standard deviation (SD). Statistical significance was determined using a threshold p-value of < 0.05.

## Results

### Constant Inflammation Enhances Oxidative Phosphorylation While Maintaining High Immunomodulatory and Low Mitotic Division Gene Expression

**Fig. 1:**
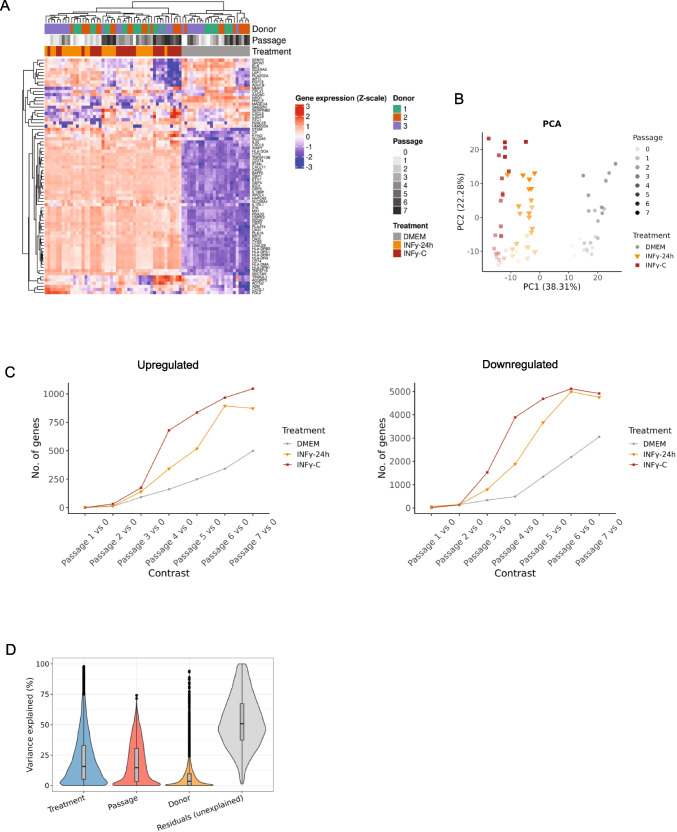
Inflammation and passage effect on data variance (**A**) Heatmap of gene expression across donors, passages, and treatment conditions. Hierarchical clustering heatmap of the top 75 most variable genes based on scaled log2-transformed expression values. Gene expression levels are Z-score normalized across samples. Columns represent individual samples, annotated by donor identity (green, orange, purple), passage number (grayscale gradient), and treatment condition (gray: DMEM, orange: IFNγ−24 h, red: IFNγ-C). Rows represent genes, clustered based on expression patterns. The color scale indicates standardized expression values, ranging from low (blue) to high (red). Clustering was performed with complete linkage and Euclidean distance. (**B**) Principal component analysis (PCA) of gene expression data. PCA was performed using the 500 most variable genes across samples. Each point represents a sample, with color denoting the treatment condition and transparency indicating the passage number. The variance explained by each PC was shown in the parentheses. (**C**) Differentially expressed genes across passages. Differential gene expression (DGE) analysis was performed using DESeq2 (version 1.46.0) to compare each passage against passage 0. The number of differentially expressed genes is shown for each treatment group, separated into upregulated (top) and downregulated (bottom) genes. (**D**) Variance partition analysis of gene expression across biological factors. Violin plots depicting the proportion of variance in gene expression explained by treatment, passage, donor, and residual (unexplained) variance. Variance components were estimated using a linear mixed-effects model (fitExtractVarPartModel from variancePartition) applied to scaled log2-transformed counts from abundant features (N = 12,000). Donor, passage, and treatment condition were modelled as random effects. The median and interquartile range are shown within each violin plot. Residual variance captures the proportion of variance not attributable to the modelled factors. Data represent variance estimates for individual genes

We analysed AD-MSCs transcriptional response to continuous IFNγ stimulation (IFNγ-C), compared to control (DMEM) and 24 h IFNγ stimulation (IFNγ−24 h) across multiple passages (P0-P7). To assess global transcriptional variability, we conducted bulk-RNA sequencing on AD-MSCs derived from 3 different donors and cultured in the 3 conditions across all passages (P0-P7). Unsupervised hierarchical clustering of the top 75 most variable genes (Fig. 1A) revealed a distinct separation between control (DMEM) and IFNγ-treated conditions, with IFNγ−24 h and IFNγ-C clustering more closely together suggesting a more similar transcriptional profile compared to DMEM.

To further dissect the main drivers of variance in our dataset, we conducted principal component analysis (PCA) (Fig. 1B). The first principal component (PC1) accounted for 38.31% of the variance, driven primarily by treatment effect, while the second principal component (PC2) accounted for 22.8%, reflecting passage-related differences. These results indicate that while IFNγ stimulation is the dominant factor influencing gene expression, the passage number also plays a significant role in shaping the transcriptome of AD-MSCs. To specifically assess the impact of passages on gene regulation under inflammatory conditions, we performed differential gene expression analysis and visualized the number of upregulated and downregulated genes across passages for each treatment (Fig. 1C). P1 and P2 displayed a similar number of regulated genes, however, as passage number increased, gene expression patterns diverged more strongly between treatments. IFNγ-C treatment, in particular, exhibited the most significant transcriptional shifts from passage 3 onward, with a markedly higher number of both upregulated and downregulated genes compared to DMEM and IFNγ−24 h (Fig. 1C). This suggests that prolonged IFNγ exposure leads to cumulative transcriptional alterations over successive passages.

To validate these findings, we applied a variance partition analysis (Fig. 1D), which confirmed that treatment condition was the most significant known factor driving transcriptional variability, followed by passage number. The residual variance indicates the presence of additional, unexplored factors influencing gene expression. These could include unknown variables like donor sex and age, adipose tissue body location and donor comorbidities.

Based on these results, we categorized passages into early (P0-P3) and late (P4-P7) groups. However, due to the variance in gene expression (Fig. 1C), late passages for the IFNγ-C treatment were defined as P3-P7. Using this classification, our analysis identified 649, 1070, and 1344 differentially expressed genes (DEGs) in DMEM, IFNγ−24 h, and IFNγ-C treatments, respectively, when comparing late versus early passages. Additionally, we identified 858 DEGs between IFNγ−24 h and DMEM, 1289 DEGs between IFNγ-C and DMEM, and 320 DEGs between IFNγ-C and IFNγ−24 h, after correcting passage effect. All comparisons were based on thresholds of |log2 FoldChange|> 1 and an adjusted *p*-value < 0.05 (Tables S1-S6).

**Fig. 2:**
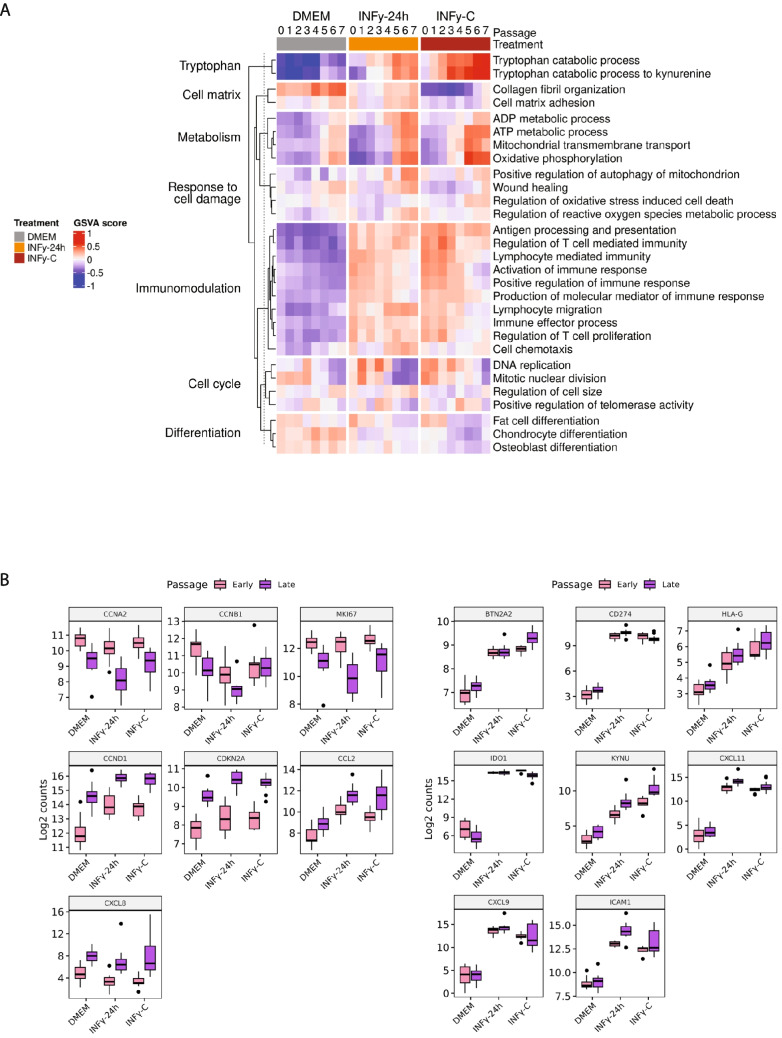
Transcriptional landscape: gene and pathway enrichment. (**A**) Heatmap of gene set variation analysis (GSVA) scores. Gene set variation analysis (GSVA) was performed using GSVA (version 2.05). For each treatment and passage, replicate values were averaged. Gene sets were first identified using gene set enrichment analysis (GSEA) and then manually curated to highlight the most relevant biological pathways. (**B**) Differential expression of cell cycle, proliferation, and immunosuppressive genes in early and late passage cells under distinct treatment conditions. Box plots display log₂-transformed gene expression counts for selected markers associated with cell cycle and proliferation (*CCNA2, CCNB1, MKI67, CCND1, CDKN2 A, CCL2, CXCL8*) *(left panel)*, and immunosuppressive functions (*BTN2 A2, CD274, HLA-G, IDO1, KYNU, CXCL11, CXCL9, ICAM1*) *(right panel)* in early (pink) and late (purple) passage cells. Cells were cultured in DMEM (control), treated with IFN-γ for 24 h (IFNγ−24 h), or subjected to continuous IFN-γ exposure (IFNγ-C). Boxes represent the interquartile range (IQR), with the median indicated as a horizontal line; whiskers extend to 1.5 × the IQR, and outliers are shown as black dots. Data are representative of independent biological replicates

We next investigated how inflammatory stimulation and passage affect AD-MSC functional properties. We performed gene set variation analysis (GSVA) across relevant biological pathways associated with metabolism, cell damage, cell cycle and immunomodulation. Figure 2A demonstrates that DMEM-treated AD-MSCs did not upregulate any immunomodulatory pathways, confirming that resting AD-MSCs lack an inflammatory transcriptional signature. In contrast, both IFNγ−24 h and IFNγ-C treatments induced upregulation of immunomodulatory pathways involved in regulation of T cell proliferation, antigen processing and presentation, and chemotaxis (Fig. 2A). Interestingly, IFNγ-C treated AD-MSCs showed a higher GSVA score for such pathways indicating a potential enhanced immunomodulatory function. Importantly, late-passage AD-MSCs exhibited reduced expression of these immunomodulatory pathways compared to early-passage cells, particularly in the IFNγ-C condition. Moreover, IFNγ-C treated AD-MSCs showed a significantly elevated GSVA score for metabolic pathways related to oxidative phosphorylation, ATP and tryptophan conversion to kynurenine, which indicates that IFNγ-C could induce a metabolic shift on AD-MSCs. Additionally, we examined GSVA scores for cell cycle-related pathways. Later-passage AD-MSCs (P5-7) exhibited lower GSVA scores for cell cycle-associated pathways, suggesting reduced activation of genes involved in proliferation.

To further characterize the transcriptional changes driving these pathway alterations, we selected key markers associated with cell cycle regulation and immunomodulation. The selected genes included *CCNB1*, *MKI67*, *CCND1*, *CDKN2 A*, *CCL2*, *CXCL8* (cell cycle related genes), and *BTN2 A2*, *CD274*, *HLA-G*, *IDO1*, *KYNU*, *CXCL11*, *CXCL9*, and *ICAM1* (immunomodulatory related genes). Consistent with our GSVA results, *CCNA2* and *MKI67* were significantly downregulated in late passages across all conditions. For *CCNB1,* IFNγ-C treatment showed no difference between early and late passage groups. In contrast, senescence-associated genes (*CDKN2 A*, *CCL2*, and *CXCL8*) were upregulated in late passages, indicating a potential transition towards replicative senesce. Notably, immunomodulatory genes remained highly expressed under IFNγ-treated conditions, suggesting that IFNγ-C promotes sustained immunomodulatory activation. While both IFNγ treatments showed clear upregulation, late passage IFNγ-C group exhibited a decreased expression of *IDO1* and *CD274.* In contrast, in this group, *KYNU, BTN2 A2 and HLA-G* displayed the highest level of upregulation, indicating differences in regulation of immunosuppressive genes.

### Constant Inflammation Impairs Early Passage AD-MSCs Proliferation and Modifies Canonical Markers Expression

**Fig. 3:**
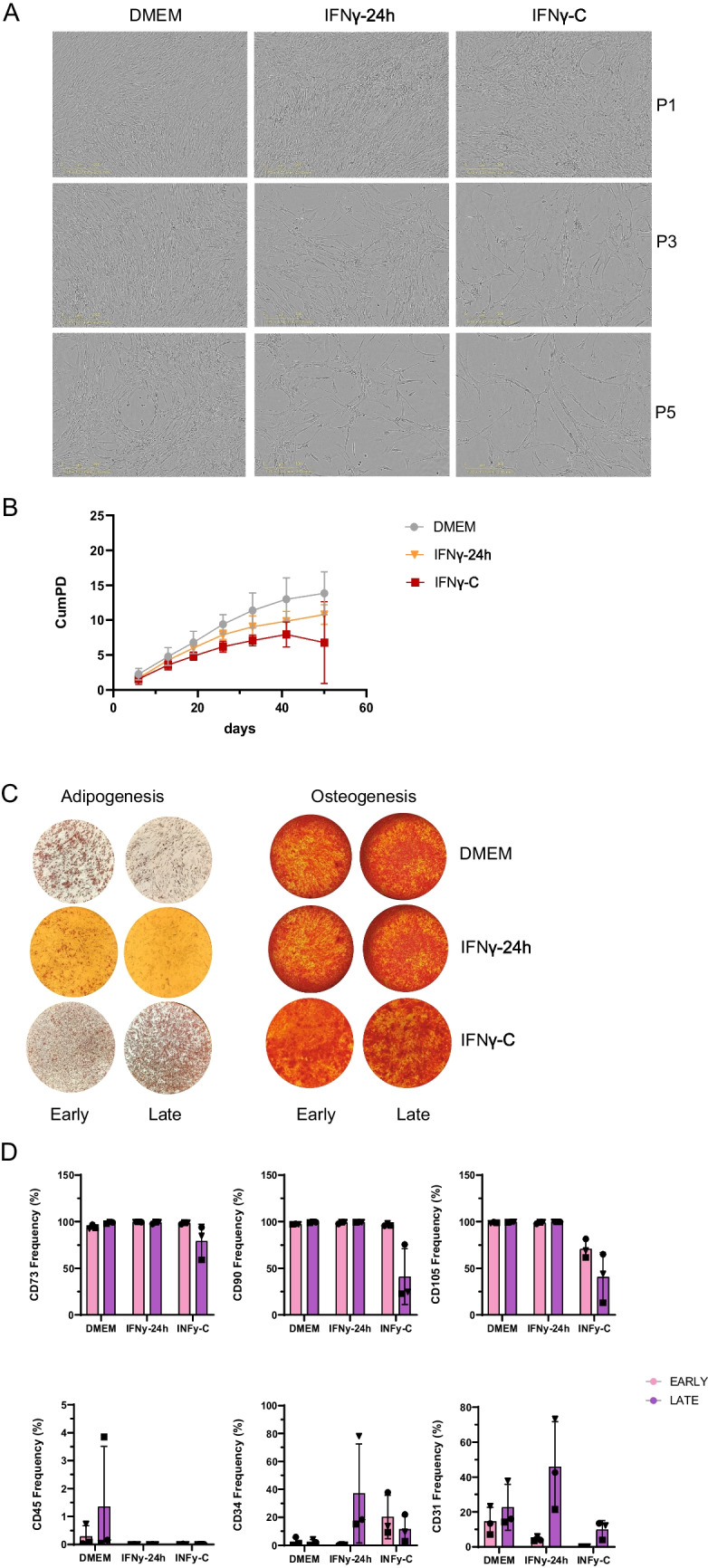
Cell proliferation, differentiation and phenotype characterisation. (**A**) Light microscopy analysis to assess AD-MSCs morphology and confluency in 24 well plates before harvesting at passage P1, P3 and P5 under DMEM, IFNγ−24 h and IFNγ-C conditions. (**B**) Cumulative population doubling (CumPD) based on cell counting prior to harvesting after each passage (P1 until P7). Each line represents a condition, DMEM (grey); IFNγ−24 h (dark grey); IFNγ-C (black). The data are presented as mean ± SD (n = 3 donors in each condition). The 2way ANOVA Tukey’s multiple comparisons test revealed significance between conditions: passage 5 DMEM vs IFNγ-C (* *p*-value < 0,05); passage 6 DMEM vs IFNγ-C (** *p*-value < 0,01; passage 7: DMEM vs IFNγ-C (*** *p*-value < 0,001). The 2way ANOVA Dunnett’s multiple comparisons test revealed significance within conditions: DMEM-P1 vs P3 (** *p*-value < 0,01); IFNγ−24 h-P1 vs P3 (* *p*-value < 0,05); IFNγ-C-P1 vs P3 (ns). (**C**) Microscopy analysis on differentiated AD-MSCs for early and late passage group across each condition (DMEM, IFNγ−24 h, IFNγ-C). Adipogenesis differentiation: AD-MSCs were stained with Oil Red O showing adipocytes in red. Osteogenesis differentiation: AD-MSCs were stained with Alizarin Red, which dyes in bright red the extracellular calcium deposits observed in osteocytes. (**D**) Flow cytometry analysis on AD-MSCs phenotypic surface markers across each treatment and for early (pink) and late (purple) groups. Representative plot show % of positive live AD-MSCs for each marker based on negative and FMO controls. Data shown are means and SD (*n* = 3). The 2way ANOVA Tukey’s multiple comparisons test revealed significance between treatments: CD90—IFNγ-C-early vs IFNγ-C-late (** *p*-value < 0,01); CD105—IFNγ-C-early vs IFNγ-C-late (** *p*-value < 0,01); CD31—IFNγ−24 h-early vs IFNγ−24 h-late (* *p*-value < 0,05)

To validate the observed transcriptional changes in cell cycle regulation, we examined AD-MSC proliferation and morphology across passages and treatment conditions (Fig. S1). Figure 3A shows that at passage 3, DMEM-treated cells reached full confluence, whereas IFNγ−24 h and IFNγ-C-treated cells exhibited lower cell densities, with IFNγ-C showing the most pronounced reduction. This indicates that IFNγ-C exposure reduces proliferative capacity.

To quantify proliferation over time, we calculated cumulative population doubling (CumPD) (Fig. 3B). CumPD is expected to increase with successive passages in proliferative cell populations. We observed a plateau in later passages, particularly in IFNγ-C-treated cells, indicating a decline in proliferative capacity. This aligns with the observed transcriptional downregulation of proliferation-related genes and enrichment of senescence pathways.

We next assessed whether IFNγ treatment affected AD-MSCs differentiation potential, a defining feature of MSCs. Figure 3C indicates that adipogenesis was impaired in late passage of IFNγ−24 h treated AD-MSCs. Contrary, IFNγ-C appeared to enhance adipogenic differentiation, as observed qualitatively by a stronger dye accumulation in late passage group (Fig. 3C). These results were unexpected and need careful consideration, but the lack of quantification of Oil Red O staining limit the interpretation of the data. Osteogenic differentiation, however, was unaffected by passage number or inflammatory conditions(Fig. 3C).

To further characterize AD-MSC identity and determine whether constant IFNγ exposure and passaging altered the defining phenotypic properties of AD-MSCs, we assessed the expression of common MSCs surface markers following ISCT guidelines (Fig. 3D, Fig. 2S). Flow cytometry analysis confirmed that nearly all cells expressed CD73, CD90, and CD105.

However, under constant inflammatory stimulation (IFNγ-C), the percentage of cells in late passages showed a marked reduction (< 50%) for CD90 and CD105 markers. Under IFNγ−24 h treatment, late passage cells showed variable CD34 expression (~ 20% in two donors and ~ 80% in one donor), with a similar but less pronounced tendency observed in early passage cells under IFNγ-C. Additionally, CD31, a marker associated with endothelial cells, was detected in ~ 20% of the cells cultured in DMEM and at similar levels in late passage cells, except for one donor showing ~ 40% positivity. Notably, late passage cells treated with IFNγ−24 h displayed a marked increase in CD31 expression (ranging from ~ 40% to ~ 70%), whereas IFNγ-C treatment resulted in lower but still detectable expression (~ 15%).

### AD-MSCs Retain Immunosuppressive Capacity on Stimulated T Cells Under Constant Inflammation

**Fig. 4:**
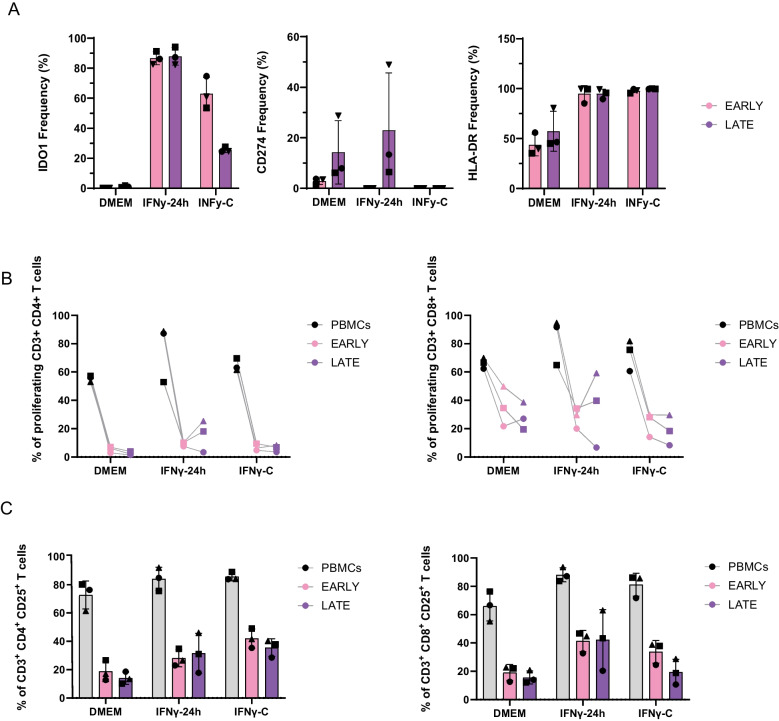
Immunosuppressive potential. (**A**) Flow cytometry analysis on AD-MSCs immunomodulatory markers across each treatment and for early (pink) and late (purple) groups. Representative plot show percentage of positive live AD-MSCs for each marker based on negative and FMO controls. Data shown are means and SD (*n* = 3). The 2way ANOVA Tukey’s multiple comparisons test revealed significance between treatments: IDO1—IFNγ-C-early vs IFNγ-C-late (**** *p*-value < 0,0001). (**B**) Flow cytometry analysis on the percentage of proliferating CD3^+^CD4^+^ T cells and CD3^+^CD8^+^ T cells stimulated with PHA in monoculture (PBMCs—dark) or in co-culture with early group AD-MSCs (pink) and late group AD-MSCs (purple). The two-way ANOVA Tukey’s multiple comparisons test revealed significance between monoculture vs early and late passages within each condition. For CD3^+^CD4^+^ T cells in all conditions PBMCs vs Early (**** *p*-value < 0000,1); PBMCs vs Late (**** *p*-value < 0000,1). For CD3^+^CD8^+^ T cells DMEM-PBMCs vs Early (* *p*-value < 0,05); IFNγ−24 h & IFNγ-C—PBMCs vs Early (*** *p*-value < 0,001); DMEM-PBMCs vs Late (** *p*-value < 0,01); IFNγ−24 h & IFNγ-C—PBMCs vs Late (*** *p*-value < 0,001). (**C**) Flow cytometry analysis on the percentage of CD3^+^CD4^+^CD25^+^T cells and CD3^+^CD8^+^CD25^+^ T cells stimulated with PHA in monoculture (PBMCs—dark) or in co-culture with early group AD-MSCs (early-pink) and late group AD-MSCs (late-purple). Representative plot show percentage of positive live T cells for each marker based on negative and FMO controls

We investigated whether the observed transcriptional changes in AD-MSCs immunomodulation translated into functional alterations in their immunosuppressive activity. To assess this, we measured the expression of immunomodulatory markers IDO1, CD274, and HLA-DR across conditions (Fig. 4A, Fig. 2S).

DMEM-treated cells exhibited no detectable IDO1 expression in either early or late passages. CD274 expression was absent in early-passage DMEM-treated cells but was detected in late passages (~ 10% of cells, except for one donor exhibiting ~ 30%). HLA-DR expression was observed in ~ 50% of DMEM-treated cells across both passages, with one donor showing variability.

IFNγ−24 h treatment induced robust IDO1 expression (~ 90% positive cells) in both early and late passages. CD274 was not expressed in early-passage IFNγ−24 h, cells but showed variable expression in late passages (~ 10%, with one donor reaching 50%). Percentage of positive cells for HLA-DR was above > 95% in both passage groups. Notably, IFNγ-C treatment resulted in modest and variable induction of IDO1 (> 50%) for early passages, a percentage that decreased to approximately 30% in late passages. Which indicates a lower IDO1 expression under constant inflammation that is exacerbated by passage effect. Additionally, CD274 remained undetectable across passages, highlighting a distinct regulatory response compared to IFNγ−24 h. HLA-DR was highly expressed (> 95%) under all IFNγ-treated conditions.

To evaluate the immunosuppressive potential of AD-MSCs, we performed a co-culture assay with PBMCs and analysed PHA-activated CD4 + and CD8 + T cell proliferation using cell trace CFSE staining and flow cytometry (Fig. 4B). By testing various AD-MSCs:PBMCs ratios, we determined that a 1:5 ratio achieved significant inhibition of T cell proliferation (Fig. S3). Notably, the extent of inhibition was strongly dependent on the number of AD-MSCs present (Fig. S3). Compared to monoculture conditions, DMEM-treated AD-MSCs significantly inhibited CD4^+^ T cell proliferation, reducing the percentage of proliferating cells from ~ 60% to < 6% in both early and late passages. IFNγ−24 h-treated AD-MSCs reduced CD4^+^ T cell proliferation from 60–90% in monoculture to 7.5–10.2% (early) and 3.4–25.4% (late), indicating a dose- and passage-dependent effect. IFNγ-C-treated AD-MSCs had a similar suppressive effect, reducing proliferation to 4–9.6% (early) and 3–8% (late). CD8^+^ T cells were less inhibited than CD4^+^ T cells. DMEM-treated AD-MSCs reduced CD8^+^ T cell proliferation from 62–70% in monoculture to 21.7–49.8% (early) and 19.5–38.6% (late). IFNγ−24 h treatment further reduced CD8 + T cell proliferation from 64–95% in monoculture to 20–34% (early) and 6.7–59% (late). IFNγ-C-treated AD-MSCs exerted stronger suppression, reducing CD8 + proliferation from 60–82% in monoculture to 14–29.8% (early) and 8–29.6% (late).

CD25 was assessed as a marker of T cell activation as well as T regulatory cell (Treg) differentiation, providing additional insight into the immunosuppressive effects of AD-MSCs under inflammatory conditions. For CD8^+^ T cells, DMEM-treated AD-MSCs reduced CD25 expression from 55–76% in monoculture to 12.6–22.7% (early) and 12.1–20.8% (late) co-cultures. IFNγ−24 h-treated AD-MSCs reduced CD25 expression from 83–93% in monoculture to 32.7–46.7% (early) and 20.4–63% (late). IFNγ-C-treated AD-MSCs followed a similar trend, reducing expression from 72–86% in monoculture to 24.5–38.8% (early) and 10.7–28.8% (late) co-cultures. For CD4 + T cells, CD25 expression was markedly reduced by AD-MSCs, decreasing from 61.5–80.2% in monoculture to 12.8–26.7% (early) and 10.1–18.6% (late) co-cultures in DMEM. IFNγ−24 h-treated AD-MSCs partially restored CD25 expression, with a milder reduction from 75–92% in monoculture to 23–34.8% (early) and 17.8–46% (late) co-cultures. IFNγ-C-treated AD-MSCs had a similar effect, reducing expression from 84–89% in monoculture to 35.4–49% (early) and 28.5–40.3% (late) co-cultures.

Together, these results indicate that IFNγ exposure modulates and maintains the immunosuppressive function of AD-MSCs.

## Discussion

The use of AD-MSCs as treatment for tissue injuries and chronic inflammatory diseases exposes them to an inflammatory microenvironment that influences their function. While AD-MSCs immunomodulatory effects on surrounding immune cells have been well documented, leading to a reduction in inflammation, the impact of this prolonged exposure on AD-MSCs themselves remains unclear. Our study provides new insights into how chronic inflammatory cues, specifically constant IFNγ stimulation, shape the molecular and functional behaviour of AD‐MSCs.

Transcriptomic analysis revealed that IFNγ-C treatment induced a shift towards oxidative phosphorylation (OXPHOS) coupled with increased ATP production (Fig. 2A), which suggests a metabolic reprogramming of AD-MSCs. This is in line with previous reports which have shown that prolonged culture or inflammatory stimuli can shift MSCs metabolism from glycolysis to OXPHOS, a change that may elevate reactive oxygen species (ROS) levels and predispose cells to mitochondrial dysfunction and replicative senescence [16–18]. Although *CDKN2 A* expression was not markedly higher under IFNγ‐C than under acute IFNγ (IFNγ‐24 h) stimulation (Fig. 2B), the reduced cell density (Fig. 3A), lower cumulative population doubling (Fig. 3B), and downregulation of proliferation‐associated genes in late passage group (*CCNA2*, *CCNB1*, *MKI67*) in IFNγ‐C-treated cells (Fig. 2B), suggest that prolonged inflammatory stress impairs AD‐MSCs proliferation. Although replicative senescence in MSCs is well recognized [19, 20], the specific contribution of chronic inflammation to accelerating senescence needs to be addressed. Our data support a model in which a constant inflammatory microenvironment may contribute to a senescence-like state, as suggested by impaired proliferation, altered morphology and canonical senesce gene expression changes. However, this senescence-like phenotype likely results from a combination of both late passaging and chronic inflammation. While our design accounts for the effects of passage, the absence of direct senescence assays, such as SA-β-gal staining, limits our ability to conclusively confirm the senescent state. Future studies incorporating SA-β-gal staining or other senescence canonical markers will be necessary to validate whether constant IFNγ exposure drives true senescence in MSCs.

While chronic exposure to inflammatory cytokines may impair the proliferative capacity of MSCs in vitro, several studies, including our findings, suggest that such preconditioning can enhance their immunomodulatory and regenerative functions in vivo [21]. This indicates a potential trade-off between expansion efficiency and therapeutic potency. From a translational perspective, short-term, controlled preconditioning with inflammatory cues (e.g., TNF-α, IFN-γ) prior to infusion may serve as a viable strategy to “license” MSCs, enhancing their in vivo functionality without severely compromising their viability or expansion potential. Furthermore, recent clinical and preclinical studies support the idea that MSCs do not need to proliferate extensively post-infusion but rather act through paracrine signalling and immunomodulation-functions that are often augmented by inflammatory priming [21].

Therefore, while constant inflammation throughout expansion could impair MSC yields, brief inflammatory preconditioning immediately before administration may preserve or even enhance therapeutic efficacy.

Cell characterisation by flow cytometry revealed that late passage AD-MSCs under constant inflammation (IFNγ‐C) show a decreased expression of CD90 and CD105, canonical MSCs markers according to ISCT (Fig. 3D). Additionally, the expression of CD34 observed in IFNγ conditions, and the variable expression of CD31 could raise concerns regarding retention of the MSCs phenotype [3, 4]. However, the persistent absence of CD45 and expression of CD73 supports the conclusion that the hematopoietic lineage is not being adopted. CD34 and CD31 is highly expressed in vascular endothelial cells and their precursors. Therefore, our data could indicate that under constant inflammation, the AD-MSCs phenotype is shifting toward an endothelial lineage, or alternatively, that there is endothelial cell contamination which becomes more prominent under these conditions. To further validate either of these hypotheses, endothelial differentiation assays should have been performed; however, the limited availability of samples constrained our ability to carry out these additional analyses. Despite some controversy in the literature regarding expression of CD34 by AD-MSCs [22, 23], a recent study examining MSCs used to treat osteoarthritis showed that MSCs increased expressed CD34 under chronic inflammation [24]. Another study showed that shear stress caused a significant upregulation of CD31 and CD34 gene and protein expression on canine MSCs [25]. Altogether, this supports our observations that sustained pro-inflammatory stimuli may alter MSCs phenotype.

Interestingly, both IFNγ‐24 h and IFNγ‐C treatments upregulated key immunomodulatory pathways, including those involved in antigen processing, chemotaxis, and T cell regulation. (Fig. 2A). The increased GSVA score for these pathways in early passage IFNγ-C-treated cells suggests that prolonged exposure to IFNγ can enhance AD-MSCs immunomodulatory functions. We monitored CD274 (PD‐L1) and IDO1 as key mediators of MSCs immunosuppression, as previous studies have demonstrated that IFNγ stimulation upregulates these molecules enhancing the immunosuppressive capacity of MSCs [26–28]. Despite *IDO1* gene being equally upregulated between both treatments and both groups (Fig. 2B), flow cytometry analysis revealed that IFNγIFNγ−24 h stimulation robustly induced IDO1 expression in nearly 90% of cells (Fig. 4A). In contrast, IFNγ‐C resulted in lower and more variable initial expression (> 50% in early passages) that decreased in later passages (Fig. 4A). Nevertheless, when comparing early passages between IFNγ‐24 h and IFNγ‐C conditions (Fig. 2S), IDO1 fluorescence intensity is higher in IFNγ‐C treated AD-MCs. Indicating a higher IDO1 expression per cell in IFNγ‐C condition compared to IFNγ−24 h (Fig. 2S). CD274 gene was also similarly upregulated between both treatments and groups, however, flow cytometry analysis only revealed a marked expression under IFNγ−24 h treatment for late passage group and no detectable expression under constant inflammation. Interestingly, despite of the lower percentage of IDO1 positive cells and the lack of CD274 expression, the overall suppressive capacity, as demonstrated by the marked inhibition of both PHA-activated CD4^+^ and CD8^+^ T cell proliferation, was maintained under IFNγ-C condition (Fig. 4B). Although several studies have underscored the critical roles of IFNγ-induced IDO1 in mediating MSCs immunosuppression, the literature does not yet clarify whether a high expression level of IDO1 is strictly necessary for optimal function. Moreover, it is plausible that additional mechanisms contribute to this maintained immunosuppressive profile. MSCs are well known to secrete a variety of paracrine factors—including TGF-β and prostaglandin E2 (PGE2), that play key roles in dampening inflammatory responses [28, 29]. These secreted factors can modulate the activity of surrounding immune cells and may act in concert with direct cell–cell interactions to sustain immunosuppression. Bulk RNA-sequencing analysis revealed that *TGFB-1* and *PTGES2* are not detected in late vs early passages within IFNγ-C treatment (Table S3) nor between treatments (Table S4-S5-S6). However, in the context of immunology other molecules known to exert an immunosuppressive effect on T cell proliferation are HLA-G and BTNA2 A [30–33]. In fact, Fig. 2B shows an increased gene expression for late passage AD-MSCs under IFNγ-C treatment compared to early passage group for both genes. Furthermore, in our co-culture system with peripheral blood mononuclear cells (PBMCs), several cell types could contribute to the overall immunosuppressive effect. PBMCs comprise not only T cells but also monocytes, natural killer (NK) cells, B cells, and dendritic cells. Monocytes, in particular, may enhance immunosuppression through mechanisms such as efferocytosis, whereby they phagocytose apoptotic MSCs and subsequently secrete anti-inflammatory mediators [34, 35]. At a minimum this would require more comprehensive investigations specifically focused on the role of monocytes in the co-culture system. As such data are not currently available, the contribution of monocytes remains speculative.

Together, these multifaceted interactions suggest that even when the proportion of IDO1-positive MSCs is lower under constant inflammatory conditions, the combined effects of paracrine signalling and crosstalk with other immune cells can preserve a robust immunosuppressive environment.

Notably, as the percentage of proliferating T cells was markedly reduced by AD-MSCs from all three culture conditions, the percentage of cells positive for CD25, a marker associated with late T cell activation and, when highly expressed on CD4^+^T cells, regulatory T cell (Treg) induction [36–40], tended to decrease on both CD4⁺ and CD8⁺ T cells in the co-cultures with IFNγ‐treated AD-MSCs, compared to monoculture (Fig. 4C). However, when comparing co-cultures across treatments, there was an overall tendency for an increased percentage of CD25‐positive cells in both IFNγ‐treated conditions, except in the case of IFNγ‐C‐treated late passage AD‐MSCs, where a decrease was observed. The reduction of CD25 expression on T cells in co-culture with AD-MSCs aligns with previous findings suggesting that AD-MSCs can inhibit CD25 expression to potentiate T cell suppression [41, 42].

Collectively, our findings indicate that constant IFNγ exposure impairs AD‐MSC proliferation. In addition, constant IFNγ drives increased heterogeneity within the AD‐MSC population. Some cells lose expression of key surface markers such as CD73, CD90 and CD105, while others acquire expression of CD34 or increase CD31 expression. Constant IFNγ exposure also led to heterogeneity in expression of IDO1, while still preserving their immunosuppressive function. While this heterogeneity might be masked in co-culture systems due to cellular redundancy, it could be critical in vivo, where local AD-MSC density is much lower and single-cell potency is important in modulating local cell interactions and immune responses.

## Supplementary Information

Below is the link to the electronic supplementary material.

Supplementary file1 (PDF 3352 KB)

Supplementary file2 (XLSX 76 KB)

Supplementary file3 (XLSX 119 KB)

Supplementary file4 (XLSX 147 KB)

Supplementary file5 (XLSX 86 KB)

Supplementary file6 (XLSX 125 KB)

Supplementary file7 (XLSX 38 KB)

## Data Availability

All the data generated in this study are included in this article and in its supplementary files.
