# Supplementary material for: Effect of Constant Inflammation on In Vitro Expanded Adipose-derived Mesenchymal Stromal Cells
Source: Stem Cell Rev Rep. 2025 Jun 5;21(6):1695–709. doi: 10.1007/s12015-025-10906-8 (PMC12356719; doi:10.1007/s12015-025-10906-8)

Fig. 1S AD-MSCs morphology across passages and conditions

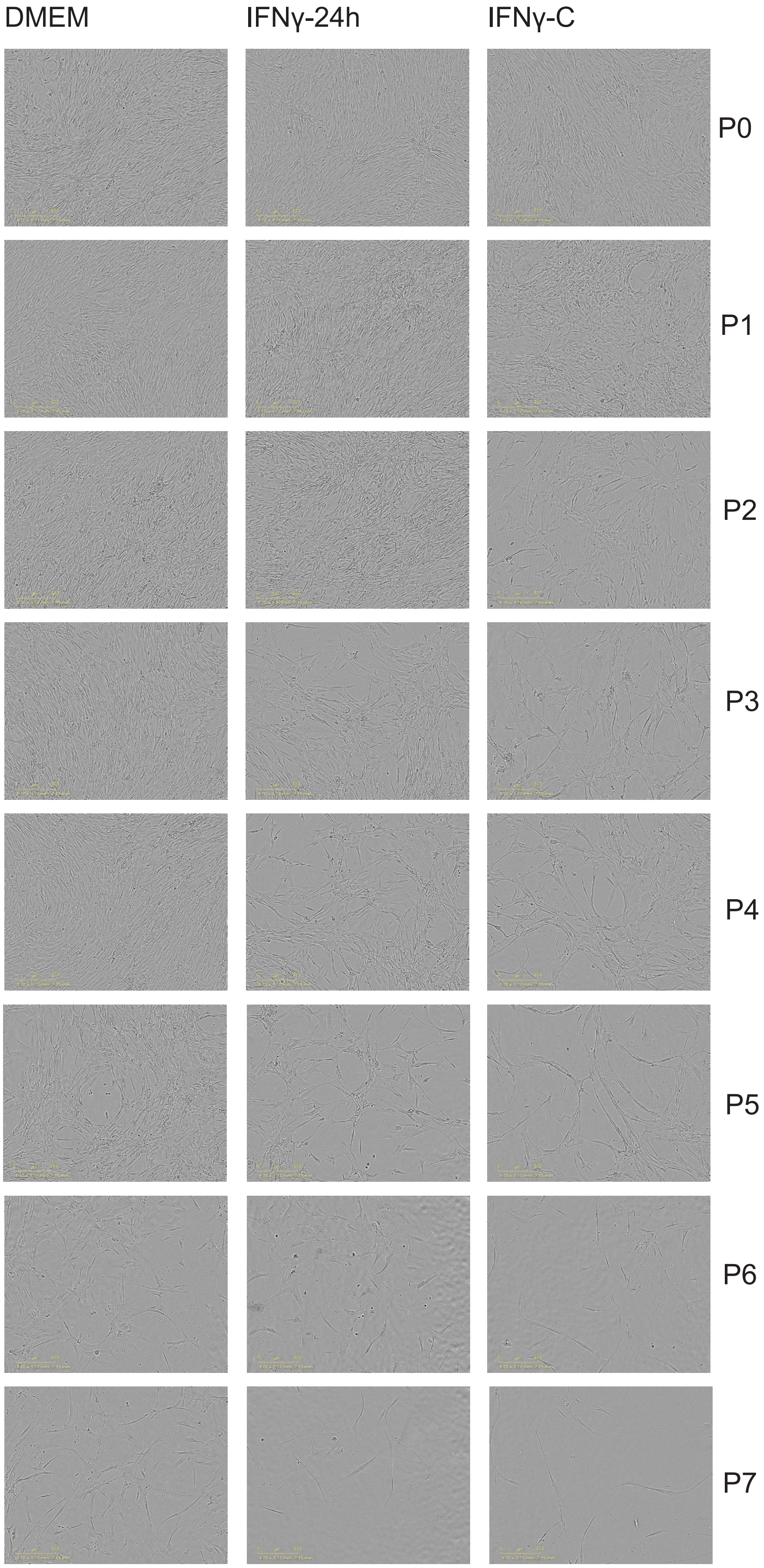

Fig. 2S Flow cytometry analysis of AD-MSCs cannonical and immunomodulatory markers

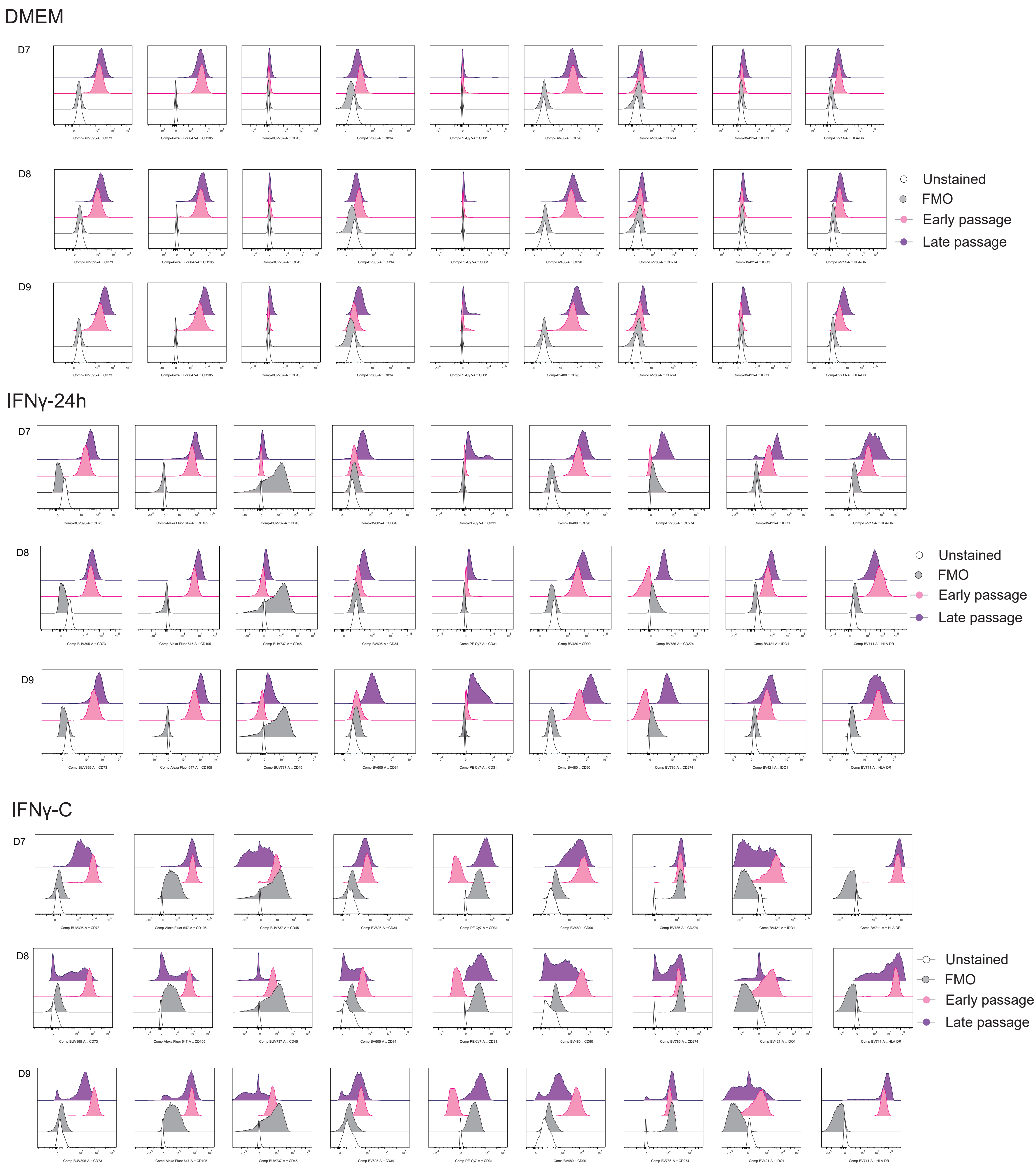

Fig. 3S Impact of AD-MSC:PBMC Ratio on T Cell Proliferation Inhibition

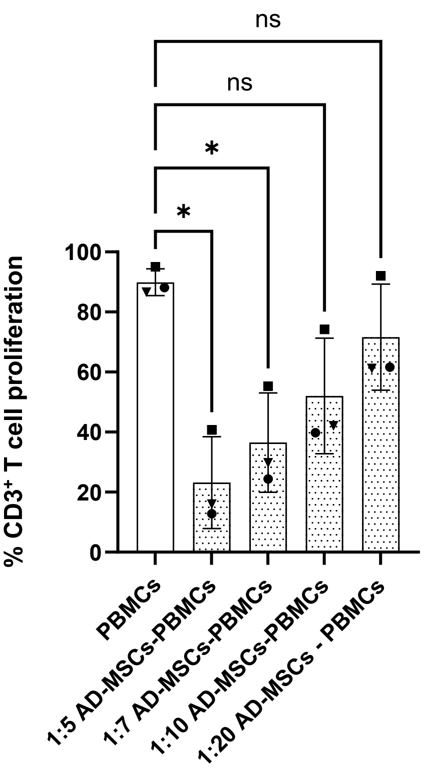

Supplement: Supplementary file 1 — Supplementary file1 (PDF 3352 KB) [file 12015_2025_10906_MOESM1_ESM.pdf]
